# Baseline arterial stiffness does not influence post‐exercise reduction in pulse wave velocity

**DOI:** 10.14814/phy2.70267

**Published:** 2025-03-04

**Authors:** Natalia S. Lima, Ronald E. Jackson, Sara R. Sherman, Brooks A. Hibner, Bo Fernhall, Tracy Baynard, Craig Crandall, Shane A. Phillips, Philip S. Clifford

**Affiliations:** ^1^ Integrative Physiology Laboratory University of Illinois at Chicago Chicago Illinois USA; ^2^ Department of Exercise & Health Sciences University of Massachusetts‐Boston Boston Massachusetts USA; ^3^ Department of Internal Medicine University of Texas Southwestern Dallas Texas USA; ^4^ Department of Physical Therapy University of Illinois at Chicago Chicago Illinois USA

**Keywords:** blood flow, compression, local arterial pressure, pulse wave velocity

## Abstract

We manipulated baseline peripheral arterial stiffness via changes in local arterial pressure with different limb positions to test the hypothesis that the magnitude of decrease in arterial stiffness post exercise/compression would be less when baseline stiffness is higher with the arm below the heart. Brachial–radial pulse wave velocity (PWV) was measured with tonometers in 19 healthy volunteers before and after 5 min of rhythmic handgrip exercise or passive forearm compressions with the arm positioned below or above the heart. Brachial–radial PWV was reduced 5 min after handgrip exercise below (10.4 ± 2.6 to 8.7 ± 2.2 m/s) and above (6.4 ± 1.3 to 5.3 ± 1.0 m/s) the heart, with no difference between positions (*p* > 0.05). PWV was also reduced 5 min after passive compressions with the arm below (10.8 ± 2.0 to 9.8 ± 2.1 m/s) and above (7.5 ± 1.4 to 5.7 ± 1.1 m/s), with no difference between positions (*p* > 0.05). Changes in local arterial pressure associated with arm position resulted in differences in baseline PWV but did not affect the magnitude of reduction in PWV with exercise or compressions. Reductions in peripheral arterial stiffness observed after rhythmic handgrip exercise and passive compressions were independent of baseline arterial stiffness.

## 1 INTRODUCTION

It is well known that chronically elevated blood pressure (i.e., hypertension) is associated with increased central and peripheral arterial stiffness (Armentano et al., 1995; Coutinho et al., 2014; Fantin et al., 2012; Ferrier et al., 2001; Kawai et al., 2015). In addition, acute changes in arterial pressure affect arterial stiffness (Bank et al., 1995; Crighton Bramwell et al., 1923; Lim et al., 2015; Nye, 1964). In particular, Zieff et al. (2019) demonstrated that changes in local arterial pressure induced by manipulating arm position affect peripheral arterial stiffness measured as blood flow, PWV, beta stiffness, compliance, or distensibility (Zieff et al., 2019). In that study, peripheral arterial stiffness was significantly greater when the arm was positioned below the heart.

Dynamic exercise acutely reduces peripheral arterial stiffness (Heffernan, Collier, et al., 2007; Heffernan, Jae, et al., 2007; Rakobowchuk et al., 2009; Ranadive et al., 2012; Siasos et al., 2016; Trachsel et al., 2019). Data from Sugawara et al. (2003) and Ranadive et al. (2012) indicate that exercise‐related reductions in peripheral arterial stiffness are due to local factors since no changes in stiffness are observed in the nonexercised limbs. Another maneuver that has local effects on peripheral arterial stiffness is passive limb compression. Heffernan, Edwards, et al. (2007) observed that passive mechanical compressions of the leg significantly reduced peripheral arterial stiffness with no changes in the control limb.

To our knowledge, it is unknown whether acute changes in baseline arterial stiffness affect the responses to an acute bout of dynamic exercise or passive mechanical compressions. Therefore, the purpose of this study was to investigate how manipulation of local arterial pressure with different limb positions affects peripheral arterial stiffness after a single bout of dynamic exercise or passive mechanical compressions. The primary hypothesis was that the magnitude of decrease in peripheral arterial stiffness post exercise or compression would be less when baseline arterial stiffness is higher with the arm positioned below the heart. Manipulating limb position below and above heart level also influences the magnitude of increase in blood flow with exercise (Egana & Green, 2005; Tschakovsky et al., 1996; Villar & Hughson, 2013) and mechanical compression (Tschakovsky et al., 1996). Since increases in flow have been associated with acute decreases in peripheral arterial stiffness after heating (Cheng et al., 2021) and reactive hyperemia (Jackson et al., 2023; Naka et al., 2006; Stoner et al., 2020), the secondary hypothesis was that the decrease in peripheral arterial stiffness post exercise or compression would be related to the magnitude of the increase in blood flow during dynamic exercise and passive mechanical compressions.

## 2 METHODS

### 2.1 Ethical Approval

The study procedures were approved by the Institutional Review Board at the University of Illinois at Chicago (2022–1129) and followed the guidelines of the Declaration of Helsinki. Written and verbal consents were obtained from each participant before they participated in this study.

### 2.2 Participants

Nineteen healthy adults (24–39 years) volunteered to participate in this study. Exclusion criteria were smoking, cardiovascular, pulmonary, metabolic, and neurological diseases. Other exclusion criteria included hypertension or hypotension, diabetes, obesity (body mass index [BMI] >35 kg/m^2^), anti‐inflammatory medication, and pregnancy. A urine‐based pregnancy test was conducted in women of childbearing age.

### 2.3 Experimental procedures

This cross‐sectional study consisted of 2 visits to the University of Illinois Chicago Integrative Physiology Laboratory. In visit 1, each participant performed rhythmic handgrip exercise with the arm below and above heart level. In visit 2, passive rhythmic compressions were performed with the participant's arm below and above heart level. Both visits occurred at the same time of day. Participants (*n* = 19) were asked to abstain from caffeine, alcoholic drinks, and exercise for at least 24 h before the study visit and fast for at least 4 h before their study visit. After signing the consent, measurement of height and weight was performed. Then participants rested quietly in the supine position for 10 min in a dark and temperature‐controlled room before instrumentation. The supine position was maintained throughout the study visit.

### 2.4 Measures

Blood pressure was continuously monitored during both visits via photoplethysmography (Finometer Pro, Finapres Medical System, Amsterdam, the Netherlands) on the middle finger of the left hand. Local arterial pressure in the forearm at the two arm positions (below and above heart level) was estimated by adjusting for the hydrostatic column differences between the heart and mid‐forearm. This was accomplished by measuring the vertical distance between the heart and mid‐forearm and converting that distance from cmH_2_O to mmHg.

Brachial‐radial PWV was assessed using 2 tonometers (Millar Instruments) placed on the proximal brachial artery and longitudinal radial artery of the right arm. The tonometer signal was sampled at 1000 Hz using PowerLab (AD Instruments, Colorado Springs, CO, USA). The tonometers acquired brachial and radial pulse waves simultaneously, and the second derivative of the pressure waves was computed on additional channels for the calculation of transit time using the “foot‐to‐foot” method. A macro was designed to automatically identify and compute the transit time between brachial and radial pressure waves. The average of at least 10 consecutive brachial‐radial pressure waves was recorded. The distance between the brachial and radial arteries was measured with a tape measure. PWV was calculated as:

$$\frac{\text{brachial}-\text{radial distance}\;\left(\text{meters}\right)}{\text{average of transit time}\;\left(\sec\right)}$$

The coefficient of variance (CV) in the PWV measurements was calculated from separate studies in 5 additional healthy subjects. The CV for PWV at resting baseline was 1.7% and after handgrip exercise was 5.0%.

Brachial blood flow was measured using an ultrasound and linear probe at 5–13 mHz (Prosound Alpha 7, Hitachi‐Aloka, Japan). Brachial diameter and flow velocity were recorded simultaneously with B‐mode and Doppler mode. Mean blood flow velocity (Vm) was obtained with the probe positioned at an insonation angle of <60°. Video recording and post‐processing were conducted using FMD Studio Cardiovascular Suite software (QUIPU, Pisa, Italy). Brachial blood velocity was acquired continuously during baseline, exercise or passive compressions, and recovery. All data were exported to an Excel spreadsheet (Microsoft, Redmond, WA, USA) for post‐analysis. Sec‐by‐sec brachial blood flow was calculated using the following equation:

$$\mathrm{BF}\;\left(\mathrm{mL}/\min^{-1}\right)=\left(V_{m}\times 60\right)\times \left(\pi \;r^{2}\right)$$

where *V*_m_ is the mean brachial artery velocity in cm/sec^−1^, multiplied by 60 to convert to cm/min^−1^, and *r* ^2^ is the brachial artery radius (cm) squared. The area under the blood flow curve (AUC) was calculated using the GraphPad Prism software (GraphPad by Dotmatics, Boston, MA, USA).

### 2.5 Protocol

After instrumentation during the first visit, participants were asked to squeeze the handgrip dynamometer 3 times with maximal force. The highest value achieved was recorded as the maximal voluntary contraction (MVC). Then participants performed rhythmic handgrip exercise at 50% of MVC for 5 min with the arms either below (~50°) or above (~50°) heart level, with the order of the two positions randomized. To maintain the appropriate angle, the subject's arm was supported on a stationary apparatus that was tilted at the appropriate angles (JawStand XP, Rockwell, Charlotte, NC, USA). Thirty min of recovery was allowed between exercise trials. The rhythmic handgrip exercise consisted of a 2‐s contraction followed by a 2‐s relaxation, with visual feedback to assist in achieving the correct amount of force. Brachial blood flow was measured at baseline, 5 min of exercise, and for 4 min post exercise (10‐min total). Brachial‐radial PWV was measured at baseline and 5, 15, and 30 min post exercise.

On a separate visit at least 24 h separated from the previous visit, a wide pressure cuff (CC17, Hokanson, Bellevue, WA) was placed on the forearm of participants for 5 min of 2 s inflation/2 s deflation cycles at 200 mmHg with a rapid cuff inflator unit (Hokanson E20, Bellevue, WA). Cuff cyclic inflations/deflations were performed with arms either below (~50°) or above (~50°) heart level in randomized order and with at least 30 min between conditions. As with the exercise trials, the arm was supported on a stationary apparatus that tilted at the appropriate angles. Brachial blood flow was measured at baseline, 5 min of compressions, and 4 min post compressions. Brachial‐radial PWV was measured at baseline and 5, 15, and 30 min post compressions.

### 2.6 Statistical analysis

PWV, brachial blood flow, and blood pressure were analysed with a two‐way (condition × time) repeated measures analysis of variance. Post‐hoc comparisons were performed using a Bonferroni correction for multiple comparisons. The significance level was set at *α* = 0.05. Data are presented as mean ± standard deviation (SD).

## 3 RESULTS

A summary of descriptive data for all 19 participants (10 females, 9 males) is presented in Table 1. One participant withdrew from the study before completion.

**TABLE 1:** Participant characteristics.

	*n* = 19	Females (*n* = 10)	Males (*n* = 9)
Age (years)	30 ± 5	31 ± 5	29 ± 5
Weight (kg)	73 ± 15	66 ± 12	80 ± 16
Height (cm)	170 ± 10	166 ± 11	174 ± 6
BMI (kg/m)	25 ± 4	24 ± 3	26 ± 4
MVC (kg)	29 ± 9	24 ± 7	35 ± 7
Race/Ethnicity	1 Black, 11 White, 2 Asian, 5 Latinx	4 White, 2 Asian, 4 Latinx	7 White, 1 Black, 1 Latinx

*Note*: Mean ± SD.

Abbreviations: BMI, body mass index; MVC, maximal voluntary contraction.

**FIGURE 1:**
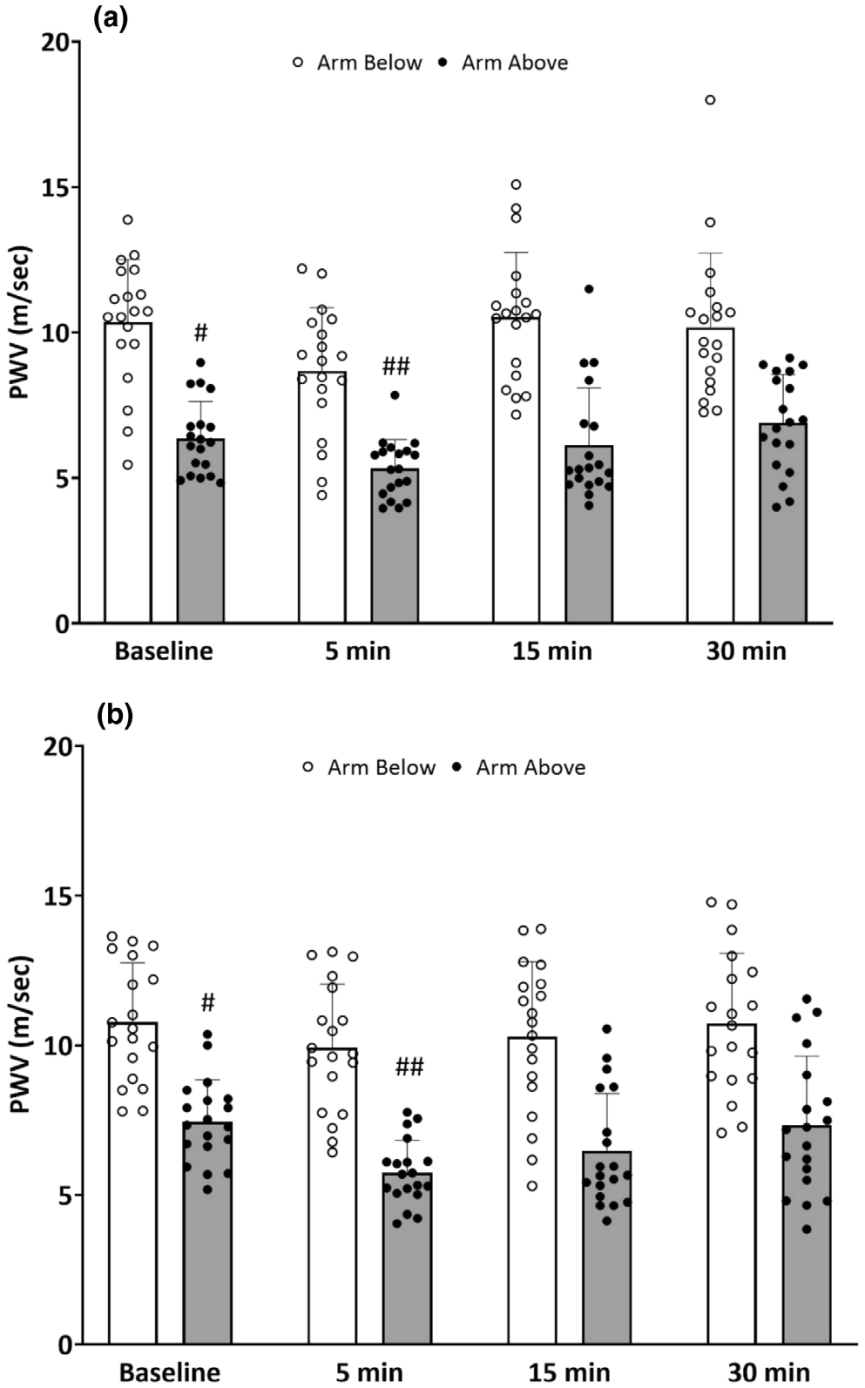
Brachial‐radial pulse wave velocity (PWV, mean ± SD) before and after 5 min of rhythmic handgrip exercise (Panel a) and passive compressions (Panel b) with the arm below and above heart level in all participants included in this study (*n* = 19). There was a main effect (*p* < 0.001) of arm position but no interaction effect (*p* > 0.05). ^#^<0.05 versus baseline with the arm below heart level. ^##^<0.05 versus baseline with the arm above heart level. Mean ± SD.

Figure 1 shows peripheral arterial stiffness measured as brachial‐radial PWV at baseline and 5‐, 15‐, and 30‐min post rhythmic handgrip exercise (Figure 1a) and passive compression (Figure 1b) with the arm below and above heart level. For both the exercise and passive compression trials, a main effect of arm position was observed for peripheral PWV (*p* < 0.001) with lower PWV when the arm was positioned above heart level and higher PWV when the arm was positioned below heart. PWV was significantly lower at 5‐min after handgrip exercise with the arm below heart (10.4 ± 2.2 to 8.7 ± 2.2 m/s; *p* < 0.001) and returned near baseline values at 15‐min (10.5 ± 2.2 m/s, *p* > 0.05) and 30‐min (10.2 ± 2.5 m/s, *p* > 0.05). PWV was also significantly lower than baseline at 5‐min post exercise with the arm above heart (6.4 ± 1.3 to 5.3 ± 1.0 m/s; *p* = 0.004) and returned to baseline at 15‐min (6.1 ± 2 m/s, *p* > 0.05) and 30‐min post (6.9 ± 1.7 m/s, *p* > 0.05). There was no position × time interaction for handgrip exercise (*p* > 0.05). Figure 1b shows peripheral arterial stiffness measured with brachial‐radial PWV before and 5‐, 15‐, and 30‐min after rhythmic passive mechanical compressions of the forearm vasculature with the arm below and above heart. After rhythmic passive mechanical compressions of the forearm, PWV was significantly reduced at 5‐min with the arm below heart (10.8 ± 2.0 to 9.8 ± 2.1 m/s; *p* < 0.001) and returned near baseline values at 15‐min (10.3 ± 2.5 m/s, *p* > 0.05) and 30‐min (10.7 ± 2.3 m/s, *p* > 0.05). PWV also decreased 5‐min after passive compressions with the arm above heart (7.5 ± 1.4 to 5.7 ± 1.1 m/s, *p* < 0.001) and returned to baseline values at 15‐min (6.5 ± 1.9 m/s, *p* > 0.05) and 30‐min post (7.3 ± 2.3 m/s, *p* > 0.05). There was no position × time interaction for passive compressions (*p* > 0.05).

Table 2 shows mean arterial pressure (MAP), systolic blood pressure (SBP), diastolic blood pressure (DBP), and pulse pressure (PP) values for rhythmic exercise and passive compressions in both arm positions. No significant differences were observed between arm positions for exercise or passive compressions for all systemic arterial pressure variables. The estimated local mean arterial pressure with the arm below the heart level was 111 ± 8 mmHg and above the heart was 62 ± 7 mmHg for the exercise visit (*p* < 0.001). During the compression visit, the estimated local mean arterial pressure was 112 ± 9 mmHg for the arm below the heart and 60 ± 8 mmHg for the arm above the heart (*p* < 0.001).

**TABLE 2:** Arterial pressure (mmHg) before and after exercise and passive compressions.

	Exercise below	Exercise above	Compressions below	Compressions above
MAP				
Baseline	88 ± 6	87 ± 7	87 ± 9	85 ± 8
5‐min	85 ± 8	88 ± 8	87 ± 9	86 ± 9
15‐min	86 ± 8	89 ± 8	87 ± 8	87 ± 9
30‐min	89 ± 11	88 ± 9	89 ± 9	85 ± 9
SBP				
Baseline	118 ± 13	120 ± 13	122 ± 13	123 ± 9
5‐min	116 ± 17	122 ± 11	123 ± 15	122 ± 10
15‐min	126 ± 17	124 ± 13	126 ± 17	124 ± 11
30‐min	128 ± 15	121 ± 14	128 ± 12	125 ± 13
DBP				
Baseline	68 ± 8	70 ± 12	70 ± 12	70 ± 7
5‐min	67 ± 10	71 ± 11	72 ± 11	69 ± 8
15‐min	74 ± 11	72 ± 11	73 ± 10	71 ± 8
30‐min	75 ± 11	70 ± 9	74 ± 8	71 ± 9
PP				
Baseline	50 ± 8	50 ± 4	47 ± 8	53 ± 8
5‐min	48 ± 10	51 ± 6	45 ± 8	53 ± 9
15‐min	51 ± 9	52 ± 6	48 ± 10	53 ± 9
30‐min	53 ± 7	52 ± 7	48 ± 8	54 ± 10

*Note*: Mean ± SD.

Abbreviations: DBP, diastolic blood pressure; MAP, mean arterial pressure; PP, pulse pressure; SBP, systolic blood pressure.

**FIGURE 2:**
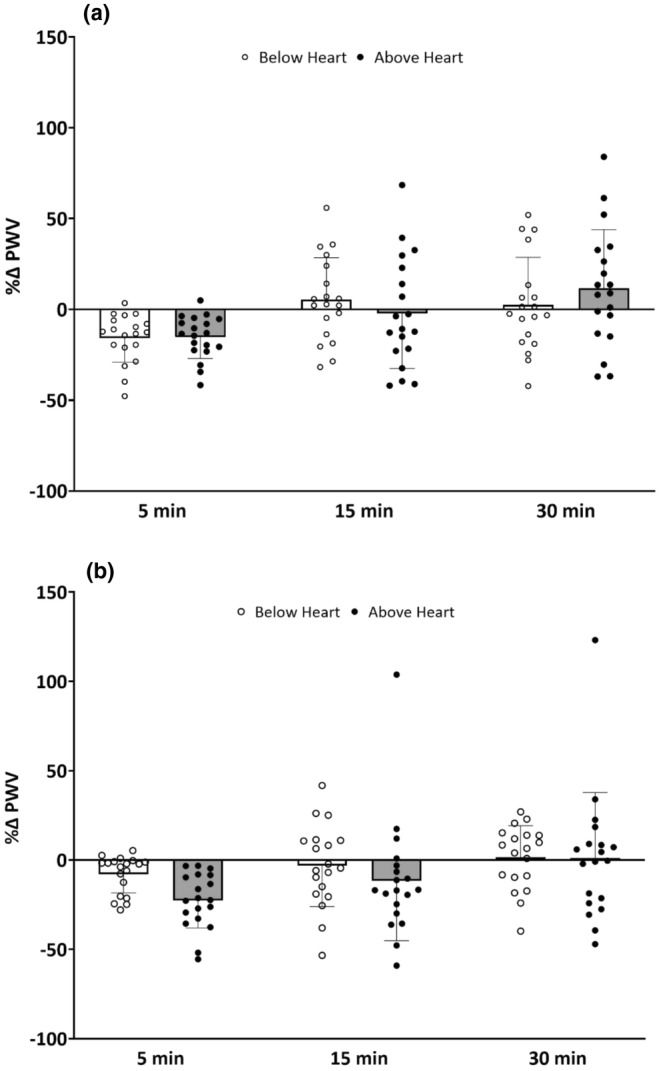
Percentage change in brachial‐radial pulse wave velocity (PWV, mean ± SD) from baseline to 5, 15, and 30 min post exercise (Panel a) or passive compressions (Panel b) with the arm below and above heart level in all 19 participants.

To compare the magnitude of change in peripheral PWV between arm below and above heart level, the percent change in PWV was calculated (Figure 2). Figure 2a shows the magnitude of change in peripheral PWV from baseline to 5‐, 15‐, and 30‐min after the rhythmic handgrip exercise with arm above and below heart level. There was no interaction effect among the conditions (*p* > 0.05). Figure 2b shows the magnitude of change in peripheral PWV from baseline to 5‐, 15‐, and 30‐min post passive compressions with the arm above and below heart level. No interaction effect was identified between conditions (*p* > 0.05).

**FIGURE 3:**
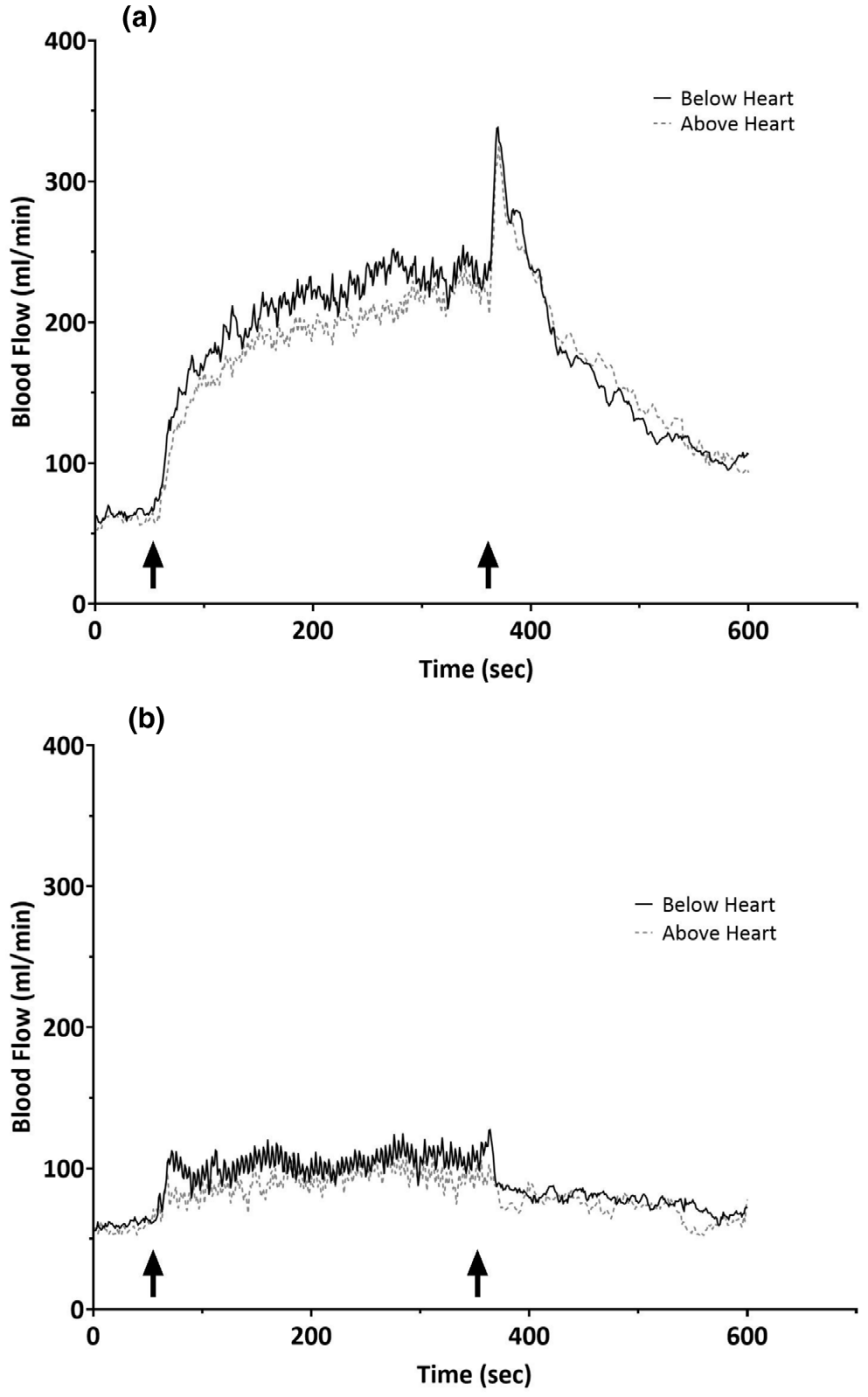
Continuous sec‐by‐sec brachial blood flow tracings from all participants from baseline, through the 5‐min intervention and 4‐min recovery (10‐min total). Panel a shows the blood flow tracings for rhythmic handgrip exercise with the arm below and above heart level. Panel b shows the blood flow tracings for the arm below and above heart level during the passive compressions of the forearm vasculature.

**FIGURE 4:**
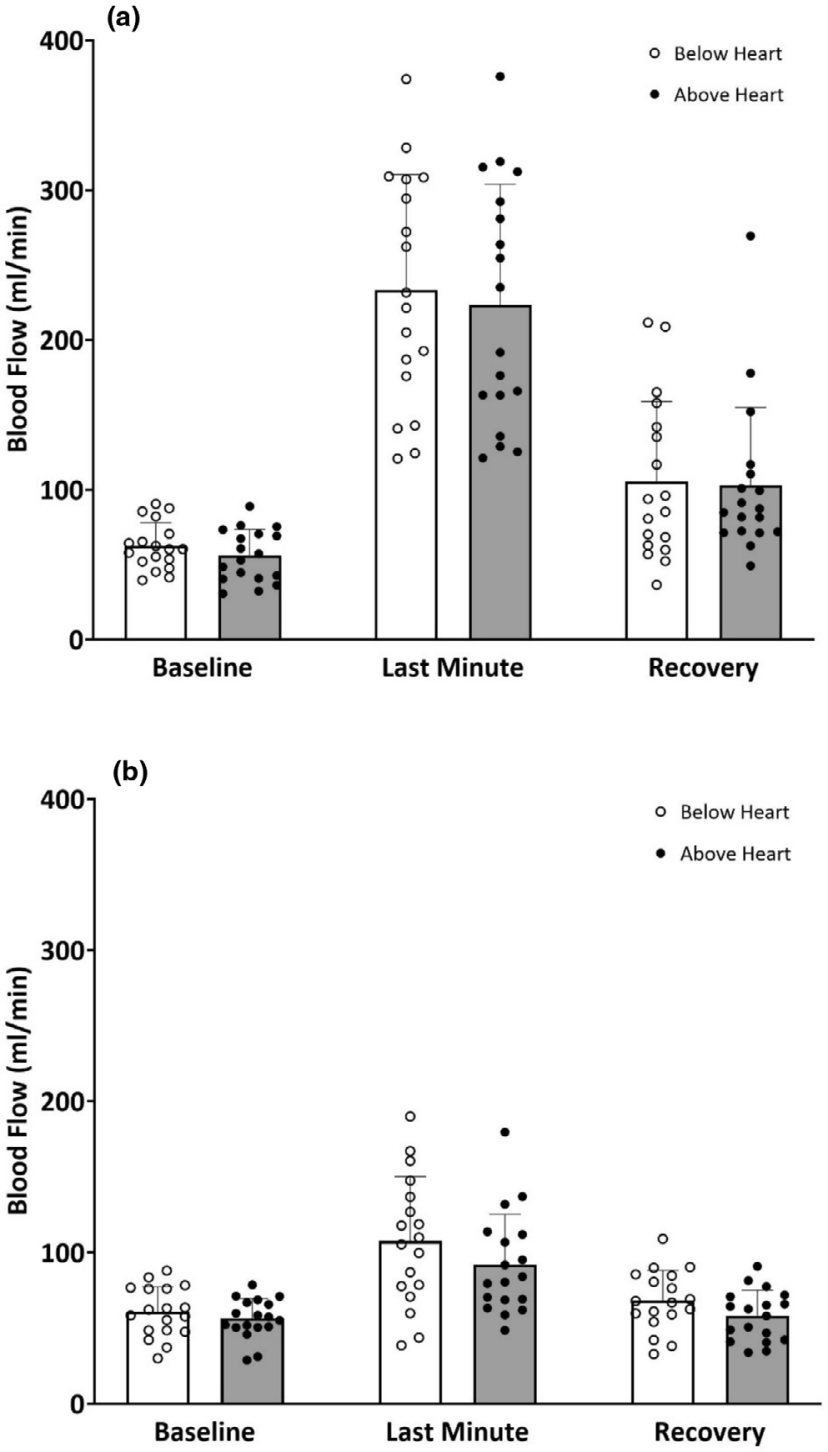
Brachial blood flow (mean ± SD) for 1‐min baseline, the last minute of intervention, and the last minute of recovery for all participants included in the study (*n* = 19). Panel a shows values for rhythmic handgrip exercise with the arm below and above heart level, and panel b shows values for passive compressions with the arm below and above heart level.

Ensemble averages of blood flow curves for all subjects during exercise and passive mechanical compressions of the forearm with the arm below and above heart level are shown in Figure 3. Exercise elicited similar patterns of increase in blood flow at the onset and during the rhythmic handgrip exercise with both arm positions (Figure 3a). A similar pattern of increase in brachial blood flow with both arm positions was also observed during passive compressions of the forearm vasculature (Figure 3b). These changes were analyzed quantitatively with 1‐min averages of blood flow at baseline, the last minute of intervention, and the last minute of recovery with the arm below and above heart level (Figure 4a,b). No statistically significant differences were observed between the arm below and above the heart for any of the time points for exercise or passive compressions (*p* > 0.05).

## 4 DISCUSSION

The purpose of this study was to investigate how manipulating baseline peripheral arterial stiffness via changes in local arterial pressure made by positioning the arm below and above heart level affects reductions in stiffness after rhythmic handgrip exercise and passive mechanical compressions. The salient finding is that the magnitude of decrease in brachial‐radial PWV after rhythmic handgrip exercise or passive compressions was unaffected by differences in baseline arterial stiffness associated with arm position. Unexpectedly, arm position also did not affect the magnitude of increase in blood flow with either intervention.

Much of the literature on pressure dependency of arterial stiffness focuses on chronic changes in stiffness, often with pharmacologic or behavioral interventions. Acute changes in stiffness occur with systemic blood pressure perturbation due to head‐up tilt, mental stress, isometric handgrip exercise, and cold pressor (Lim et al., 2015) or whole‐body postural changes (Schroeder et al., 2017). Zieff et al. (2019) employed a more isolated effect of local arterial pressure on peripheral arterial stiffness using changes in limb position. Their results demonstrated that an increase in local arterial pressure caused by lowering the arm below heart level increased peripheral arterial stiffness measured in the brachial artery. It is important to point out that they employed a single‐point method with ultrasound, while the two‐point PWV with tonometers remains the gold standard method (Boutouyrie et al., 2021; Chirinos, 2012). Indeed, the same group has shown that single‐point PWV does not directly reflect regional 2‐point PWV (Fryer et al., 2020). To our knowledge, the current study is the first to demonstrate that local arterial pressure directly influences peripheral arterial stiffness assessed with two‐point measurement of peripheral PWV with tonometers.

Our results demonstrate a significant reduction in PWV 5 min after rhythmic handgrip exercise at 50% MVC. This is consistent with the body of literature showing a reduction in peripheral arterial stiffness after an acute bout of dynamic exercise (Campbell et al., 2011; Doonan et al., 2011; Fryer et al., 2021; Heffernan, Collier, et al., 2007; Heffernan, Jae, et al., 2007; Kingwell et al., 1997; Okamoto et al., 2018; Ranadive et al., 2012; Sugawara et al., 2003). The results of the current study also demonstrated a significant reduction in PWV 5 min after passive mechanical compressions ceased, which is consistent with a previous study by Heffernan, Edwards, et al. (2007). The novel finding in the current study is that baseline arterial stiffness did not affect the magnitude of the reduction in PWV after exercise or passive compressions. Changes in local arterial pressure associated with arm position had the anticipated effect on baseline brachial–radial PWV, but the magnitude of the decrease in PWV associated with exercise or mechanical compressions was independent of the baseline values.

It is noteworthy that the decrease in peripheral arterial stiffness after acute exercise or passive compressions is exclusively observed in the involved limbs (Heffernan, Edwards, et al., 2007; Ranadive et al., 2012; Sugawara et al., 2003). Thus, it seems that a local factor must contribute to the responses. We reasoned that one relevant local factor is the increase in limb blood flow with exercise or passive compressions. Since previous studies have observed decreases in peripheral arterial stiffness after increases in blood flow were elicited by heat (Cheng et al., 2021) or by reactive hyperemia (Naka et al., 2006; Stoner et al., 2020), it seemed plausible that increases in blood flow may play a role in the reduction of peripheral arterial stiffness after acute exercise. Manipulation of limb position has been shown to influence the magnitude of change in blood flow during exercise (Egana & Green, 2005; Tschakovsky et al., 1996; Villar & Hughson, 2013). A single contraction of the forearm produced a higher peak blood flow when the arm was below heart level compared to above (Tschakovsky et al., 1996). A series of rhythmic calf contractions showed higher blood flow when the legs were below heart level (Egana & Green, 2005; Villar & Hughson, 2013). Similarly, passive compressions of the forearm elicited a higher blood flow response when the arm was below heart level (Tschakovsky et al., 1996) and reactive hyperemia produced a higher blood flow response when the arm was below heart level (Jasperse et al., 2015). Based on these previous findings, we postulated that rhythmic handgrip and passive compression would produce higher blood flow when performed below heart level. However, our results showed no difference in the magnitude of brachial blood flow change between below versus above heart level. It is difficult to provide an adequate explanation for this inconsistency. One potential explanation may be related to the timing of contractions and compressions. Tschakovsky et al. (1996) performed 1‐s/2‐s contraction/relaxation and inflation/deflation cycles for 1 min, whereas we performed 2‐s/2‐s contraction/relaxation and inflation/deflation cycles for 5 min. It is possible that the comparable reduction in PWV in the two arm positions in the current study is related to the similarity in the brachial blood flow responses.

The fact that differences in baseline arterial stiffness did not affect the magnitude of decrease in brachial‐radial PWV after rhythmic handgrip exercise has potential implications for hypertensive individuals who have chronically elevated arterial stiffness (Coutinho et al., 2014). Exercise should be effective in acutely lowering arterial stiffness in their exercising limbs independent of their blood pressure. This speculation should be verified in future studies.

There are some limitations to this study. Although both male and female volunteers were included, the study was not adequately powered to examine sex differences. All the subjects were young and healthy. No older individuals or hypertensive individuals were included. Sex differences, aging, and hypertension would be good topics for future studies. We must acknowledge that local arterial pressure in the experimental limb was estimated rather than directly measured. This calculation is rather straightforward and has been used by other laboratories (Jasperse et al., 2015; Tucker et al., 2020; Walker et al., 2007).

## 5 CONCLUSION

Data from this study confirm that local arterial pressure affects baseline peripheral arterial stiffness such that baseline brachial‐radial PWV was higher when the arm was below the heart and lower when the arm was above the heart. In contrast to our hypothesis, the data also demonstrate that arm position did not affect the magnitude of decrease in peripheral arterial stiffness measured with brachial‐radial PWV after rhythmic handgrip exercise or passive compressions. Importantly, the reduction in peripheral PWV after rhythmic handgrip exercise or passive compressions was independent of arm position and local arterial pressure‐mediated changes in baseline arterial stiffness.

## FUNDING INFORMATION

There was no external funding.
